# Antihypertensive Drug Use and New-Onset Diabetes in Female Patients with Coronary Artery Disease: A Population-based Longitudinal Cohort Study

**DOI:** 10.1097/MD.0000000000001495

**Published:** 2015-09-11

**Authors:** Yi-Sheng Liou, Hung-Yi Chen, Lyun Tien, Yi-Sian Gu, Gwo-Ping Jong

**Affiliations:** From the Department of Family Medicine and Geriatrics, Taichung Veteran General Hospital, and School of Public Health, National Defense Medical Center, Taipei, Taiwan, ROC (Y-SL); Department of Pharmacy, China Medical University Hospital and China Medical University Beigang Hospital, Taichung and Beigang Township, Yunlin County, Taiwan, ROC (H-YC, Y-SG); Central Region Branch, Bureau of National Health Insurance, Taichung, Taiwan, ROC (L-T); and Division of Internal Cardiology, Chung Shan Medical University Hospital and Chung Shan Medical University, and Basic Science, Central Taiwan University of Science and Technology, Taichung, Taiwan, ROC (G-PJ).

## Abstract

Antihypertensives have been linked to new-onset diabetes (NOD) and different classes of antihypertensives may alter the risk for the development of NOD; however, the effect of different antihypertensives on the development of NOD in women with hypertension and coronary artery disease (CAD) has not been well studied. The purpose of this study is to investigate the association between usage of different antihypertensive drugs and the development of NOD in female patients with hypertension and CAD.

Data in this retrospective cohort study were obtained from claim forms submitted to the Taiwan Bureau of National Health Insurance in central Taiwan during the period 2006–2011. We estimated the odds ratios (OR) to approximate the relative risk of NOD development associated with antihypertensive drug use.

Of the 20,108 female patients with CAD at baseline, 2288 patients developed NOD during the 6-year follow-up. Subjects treated with angiotensin-converting enzyme (ACE) inhibitors (OR, 0.92; 95% confidence interval [CI], 0.84–1.00), angiotensin receptor blockers (OR, 0.92; 95% CI, 0.82–0.99), and alpha-blockers (OR, 0.88; 95% CI, 0.79–0.98) in the adjusted analyses had greater reductions of the risk than among nonusers. Patients who took diuretics (OR, 1.10; 95% CI, 1.01–1.20), beta-blockers (OR, 1.12; 95% CI, 1.04–1.21), and calcium channel blockers (OR, 1.10; 95% CI, 1.02–1.18) were at high risk of developing NOD than nonusers. Vasodilators were not associated with risk of NOD.

We conclude that women with hypertension who take ACE inhibitors, angiotensin receptor blockers, and alpha-blockers are at lower risk of NOD and that use of diuretics, beta-blockers, and calcium channel blockers was associated with a significantly increased risk of developing NOD during the 6-year follow-up.

## INTRODUCTION

Diabetes mellitus is a major risk factor for coronary heart disease and contributes significantly to cardiovascular morbidity and mortality both in men and women.^1,2^ Each year more women than men die from coronary artery diseases (CAD) including myocardial infarction and sudden cardiac death. Studies have shown that the prevalence of diabetes, especially new-onset diabetes (NOD), is increasing in women worldwide.^3,4^ A number of prospective trials on antihypertensive drug use have investigated whether these agents are associated with the development of NOD in hypertensive patients.^5–10^ Although the majority of studies found that cardiovascular risk is higher when diabetes and hypertension coexist than when the two conditions stand alone in women, data from these studies are limited because the majority of epideminological studies on NOD have focused on men or on Caucasian populations.^10–12^ In addition, most studies have investigated only a single class of antihypertensive agent, with angiotensin receptor blockers (ARBs) being the most commonly studied.^12,13^ Thus, it is not completely clear whether certain antihypertensive drug classes are associated with higher risk for NOD than other antihypertensive drug classes in female patients with CAD.

In this retrospective cohort study, we explored the relationship between antihypertensive drugs (diuretics, beta-blockers, calcium channel blockers [CCBs], alpha-blockers, vasodilators, angiotensin converting enzyme [ACE] inhibitors, ARBs) and the development of NOD in female hypertensive patients with CAD.

## METHODS

### Subjects

Data were obtained from claim forms provided to the central regional branch of the Bureau of National Health Insurance (BNHI) in Taiwan during the period 2006 through 2011. The BHNI stores information from claim forms in 2 tables: a visit table and a prescription table. Visit tables contain information regarding patient identification numbers, sex, age, 3 diagnostic codes, and medical expenditures, as well as information pertaining to the medical institutions and attending physicians. The prescription table lists the quantity and expenditure for all drugs, operations, and treatments. We summarized the claim records of each patient into 1 record.

### Study Design

**FIGURE 1:**
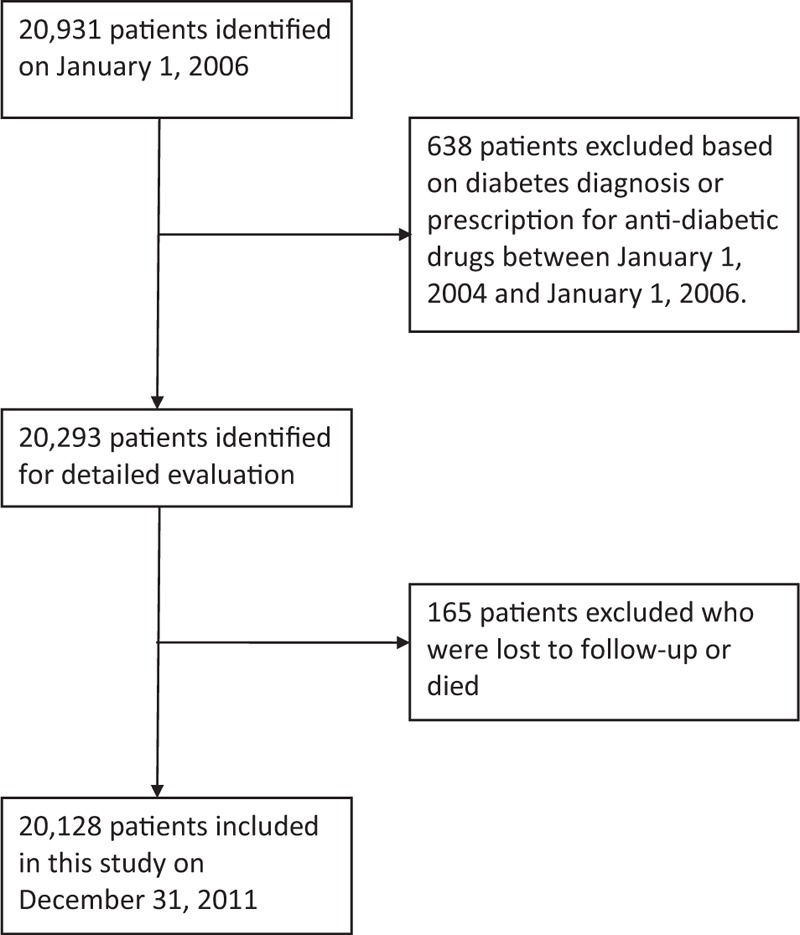
Flowchart of selection of patients for the inclusion in this study.

At baseline (January 1, 2006), we excluded 638 hypertensive patients (International Classification of Diseases, Ninth Revision Clinical Modification (ICD-9-CM) codes 401–405) and CAD (ICD-9-CM codes 410–414) because they had diabetes diagnosis (ICD-9-CM code 250) or prescription for antidiabetic drugs between January 1, 2004 and January 1, 2006. A total of 20,293 hypertensive patients without diabetes were included in the study at baseline. Patients were followed-up from study entry until the NOD diagnosis, death, or end of follow-up, whichever occurred first. The end of the follow-up period was December 31, 2011. The primary study outcome was the development of NOD, which was defined as the first time that a diabetes code or antidiabetic prescription appeared in the outpatient claim records. During the 6-year follow-up, we excluded 165 patients who were lost to follow-up or died. Finally, 20,128 patients were enrolled in the analysis (Figure 1). Patients were grouped into 1 of the following 7 mutually exclusive exposure groups defined by *ever* use of (1) diuretics, (2) beta-blockers, (3) CCBs, (4) alpha-blockers, (5) ACE inhibitors, (6) ARBs, and (7) vasodilators.^14^ In Taiwan, these antihypertensive drugs are available only by prescription. This study was approved by the Institutional Review Board of the Armed Forces Taichung General Hospital (No. 97018).

### Statistical Analysis

Data were described with means and standard deviation for normally distributed variables and with frequencies and percentages for categorical variables. The unpaired Student *t* test or the chi square test were examined for the differences between the NOD group and the non-NOD group in the distribution of demographic characteristics, comorbidities, and concurrent medications. We compared both the drug use and nonuse subjects in order to find out which drug classes might increase or decrease the probability of developing NOD with the Cox regression model, adjusting for age, comorbidities, and concurrent medication.^15^ All data management and OR calculations were done using the Statistical Analysis System (SAS) software for Windows (version 9.1; SAS Institute, Cary, NC). A *P* value < 0.05 was considered statistically significant.

## RESULTS

### Population Characteristics

Of the 20,128 eligible subjects, 2288 (11.4%) developed NOD during the period 2006–2011. The mean age of NOD patients was 64.8 *+* 13.4 years and that of non-NOD patients was 64.3 ± 13.1 years. There were no significant differences in age between the 2 groups of patients (*P* = 0.93) (Table 1).

**TABLE 1:**
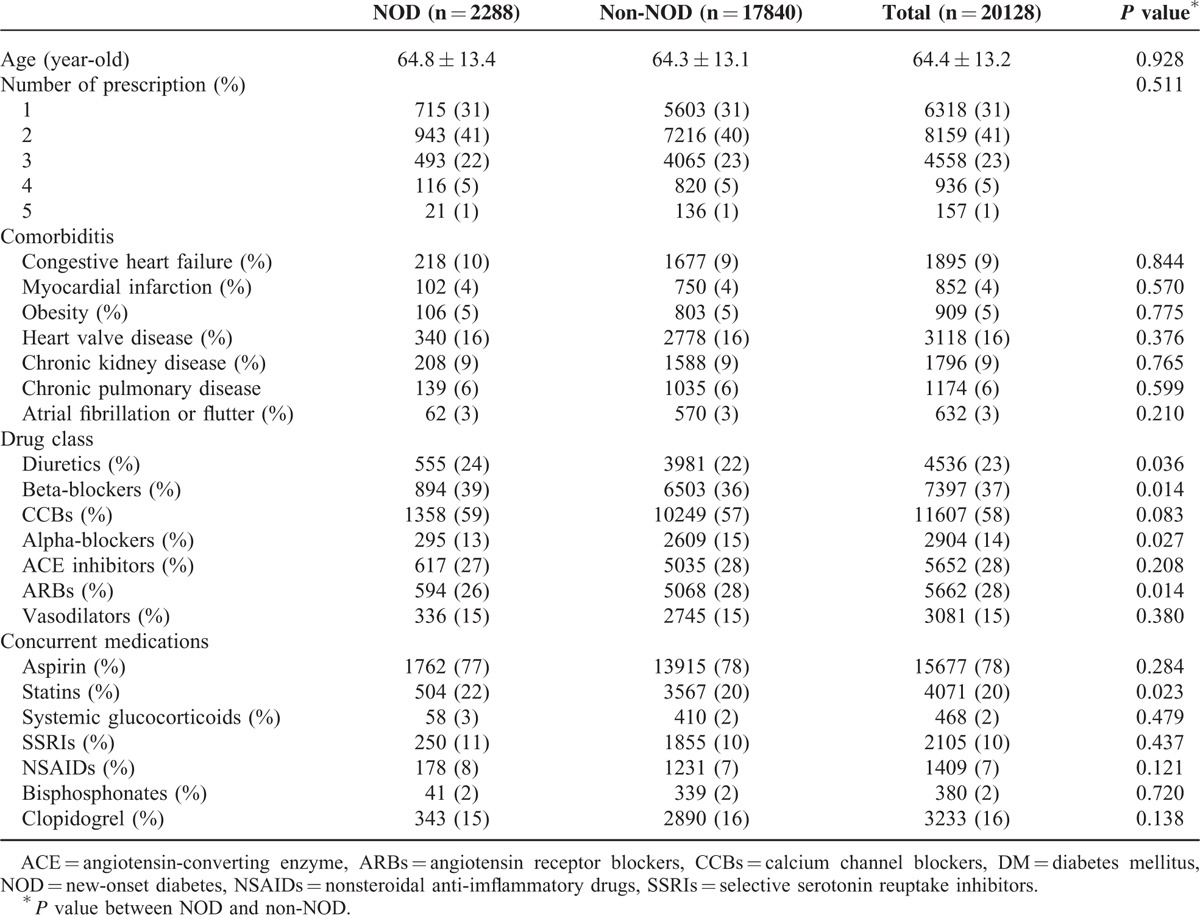
Baseline Characteristics of All Patients

In addition, 31% (6318) of the patients took only 1 drug class, 41% (8159) took 2 drug classes, 23% (4558) took 3 drug classes, 5% (936) took 4 drug classes, and 1 % (157) of patients took 5 drug classes (Table 1). At baseline, there were no significant differences in prevalence of congestive heart failure, myocardial infarction, obesity, heart valve disease, chronic kidney disease, chronic pulmonary disease, and atrial fibrillation or flutter between the 2 groups of patients. Nearly 58% of subjects took CCBs, 37% of subjects took beta-blockers, 28% of subjects took ACE inhibitors and ARBs, 23% of subjects took diuretics, 15% of subjects took vasodilators, and 14% of subjects took an alpha-blocker. NOD subjects took more statins than subjects without NOD, but there were no significant difference in usage of aspirin, systemic glucocorticoids, selective serotonin reuptake inhibitors, nonsteroidal anti-inflammatory drugs, bisphosphonates, or clopidogrel between the 2 groups.

### Cox Survival Analysis Adjusted for Age, Comorbidities, and Concurrent Medication

Users of diuretics (OR, 1.10; 95% confidence interval [CI], 1.01–1.20), beta-blockers (OR, 1.12; 95% CI, 1.04–1.21), and CCBs (OR, 1.10; 95% CI, 1.02–1.18) were at significantly higher risk of developing NOD than nonusers after adjusting for age, comorbidities, and concurrent medication usage (*P* < 0.05). Users of Alpha-blockers (OR, 0.88; 95% CI, 0.79–0.98), ACE inhibitors (OR, 0.92; 95% CI, 0.84–0.90), and ARBs (OR, 0.92; 95% CI, 0.82–0.99) were at a lower risk of developing NOD than nonusers. Vasodilators were not associated with risk of developing NOD (*P* > 0.05) (Table 2).

**TABLE 2:**
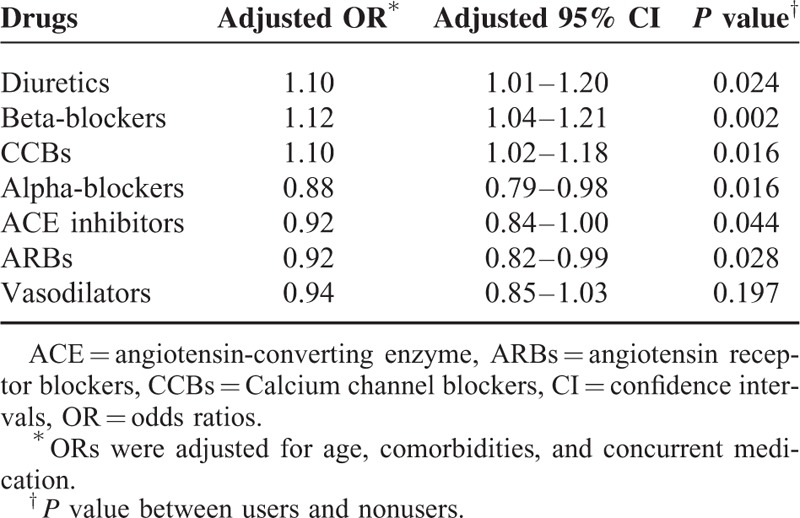
Incidence of ORs with 95% CIs for New-Onset Diabetes According to Prescriptions for Antihypertensive Drugs Compared with Nonuser Subjects

## DISCUSSION

In this population-based longitudinal study, we found that ACE inhibitors, ARBs, and alpha-blockers were independently associated with a decreased risk of developing NOD and that diuretics, beta-blockers, and CCBs were independently associated with an increased risk of developing NOD in women with hypertension and CAD in central Taiwan. Vasodilator usage was not associated with NOD development.

Previous studies have demonstrated that diuretics accelerate the development of NOD in patients with hypertension.^16,17^ It has been suggested that diuretic therapy has been associated with impaired insulin release through depletion of serum potassium and increase hepatic insulin resistance, resulting in continued hepatic glucose production despite high insulin levels.^18,19^ Our data are consistent with the results from a large randomized clinical trial showing an increased risk for NOD in individuals taking a diuretic as compared to placebo.^20^ Similarly, some observational studies have indicated that women taking diuretics have a 10% to 30% higher risk of developing NOD than those not taking diuretic drugs.^21,22^ Taylor et al reported a significant 20% increased risk of developing NOD in older women and a 45% increased risk of developing NOD in younger women after diuretic treatment respectively.^22^ In contrast, Padwal et al found no association between the use of thiazide diuretics and NOD.^23^ However, their study had a mean follow-up period of <1 year and may have lacked statistical power.^24^

Beta-blockers may worse insulin resistance through reduced cardiac output and peripheral glucose ulitization.^19^ Therefore, recent evidence suggests that long-term use may increase the risk of NOD.^19^ Our finding that beta-blocker usage is associated with an increased risk of developing new onset diabetes is similar to that reported in previous studies.^22,25^ However, other studies have reported that beta-blockers have a neutral effect on risk of NOD in patients with hypertension.^20,23^ In the Nateglinide and Valsartan in Impaired Glucose Tolerance Outcomes Research (NAVIGATOR) trial, which included 9306 patients, the authors reported that there was no association between beta-blocker use and NOD.^20^ The high risk for diabetes mellitus (impaired glucose intolerance) and the relatively small sample size (5640 patients) in that study may partially explain the discrepancy between our findings and the findings reported in the NAVIGATOR trial.

Calcium channel blockers are generally considered to have a neutral effect on the development of NOD.^22,26,27^ Many studies have indicated that CCBs are associated with a greater risk of NOD than ACE inhibitors and ARBs but a lower risk of NOD than beta-blockers and thiazide diuretics. Our finding that calcium channel blockers increased the risk of NOD is similar to that reported in the Nurses’ Health Study (NHS) I, which found that older women who took oral calcium channel blockers were at higher risk of developing NOD than women taking placebo.^22^

ACE inhibitors or angiotensin receptor blockers may improve insulin sensitivity secondary to kinin, prostaglandins or nitric oxide accumulation, and increased peripheral blood flow to skeletal muscle.^19^ Thus, many studies have shown that blockers of the renin angiotensin system (ACE inhibitors and ARBs) reduce the risk of developing NOD when compared to placebo.^28–31^ In the present study, both ACE inhibitors and ARBs were found to have protective effects against developing NOD compared to placebo during antihypertensive therapy. A similar finding was reported in the Heart Outcomes Prevention Evaluation (HOPE)^28^ and NAVIGATOR^20^ trials. However, the Diabetes Reduction Assessment with Ramipril and Rosiglitazone Medication (DREAM) trial failed to show a statistically significant reduction in NOD with the ACE inhibitor ramipril versus placebo in patients with impaired fasting glucose.^29^ The lack of hypertension as an inclusion criterion and the relatively short follow-up period (3 years) in the DREAM trial might explain why no significant differences in NOD were detected between the 2 groups.

We found that the incidence of NOD was significantly lower among patients who took Alpha-blockers. Previous studies have consistently demonstrated that alpha-blocker classes of antihypertensive medications have protective effects on carbohydrate and lipid metabolism because alpha-blockers may promote peripheral vasodilation and improve insulin sensitivity and glucose uptake.^32^ However, to the best of our knowledge, no studies have investigated the relationship between alpha-blocker usage and risk of developing NOD in women with hypertension and CAD.

In the present study, vasodilators were found not to be associated with NOD in patients with hypertension. To the best of our knowledge, no studies have evaluated the relationship between vasodilators and NOD.^17,22^

Our study also has some limitations. First, our data were derived from a health insurance database. Therefore, actual blood sugar levels and some important confounding variables such as body mass index of patients, family history, and smoking status were not available. However, because the data we used were population-based data, we assumed that there were no differences among the 7 antihypertensive groups. Second, the process of insulin resistance in this study of patients who developed NOD must have started many years before the diagnosis and it might have coexisted with the process of hypertension for which antihypertensives were used. The exposure and follow-up period of our study was relatively long and the patients were not new users but current users. In this situation, the cause and effect relationship between antihypertensive agents and NOD development cannot be determined in this study. Third, all diagnoses of diabetes mellitus were based on physician reporting in central Taiwan only; therefore, it is not clear how our findings can be generalized to patients in different areas.

## CONCLUSIONS

Our results suggest that ACE inhibitors, ARBs, and alpha-blockers reduce the risk of developing NOD. Our findings could have practical clinical applications for strategies to prevent adverse outcomes in women with hypertension and CAD.
